# Individual and Combined Effects of Bisphenol A and Lithium on the Regenerative Capacity of the Polychaete *Hediste diversicolor*

**DOI:** 10.3390/toxics14090832

**Published:** 2026-09-20

**Authors:** Valéria Giménez, Etelvina Figueira, Adília Pires

**Affiliations:** Center for Environmental and Marine Studies (CESAM), Department of Biology, University of Aveiro, 3810-193 Aveiro, Portugal; valeriacgimenez@ua.pt (V.G.); efigueira@ua.pt (E.F.)

**Keywords:** benthic invertebrates, regeneration, bisphenol A, lithium, mixture toxicity

## Abstract

Bisphenol A (BPA) and lithium (Li) are emerging contaminants widely detected in aquatic environments, yet their effects on regenerative processes remain poorly understood. This study evaluated the effects of BPA, Li, and their mixture on the regenerative capacity of the estuarine polychaete *Hediste diversicolor*, an important bioturbator in coastal ecosystems. Organisms were exposed for 21 days to BPA (0–135 μg/kg), Li (0–78.13 mg/kg), and BPA + Li mixtures under controlled laboratory conditions. Regeneration was assessed by determining the number of regenerated segments and the percentage of body width following posterior amputation. Results showed that BPA reduced regenerative capacity across all treatments, with the strongest effects observed at 5 and 45 μg/kg. Li exposure also decreased regeneration, particularly at 12.5 mg/kg. Combined exposure also resulted in reduced regenerative capacity, with the strongest effect observed at 12.5 mg/kg Li + 45 μg/kg BPA. Regeneration in mixture-exposed organisms was generally comparable to that observed following individual exposures. Notably, organisms exposed to 12.5 mg/kg Li and 5 μg/kg BPA individually had lower regenerative capacity than the corresponding mixture treatment, suggesting that co-exposure did not exacerbate the effects observed under individual exposures. Overall, BPA and Li negatively affect regeneration in *H. diversicolor*, highlighting regeneration as a sensitive endpoint for ecotoxicological assessment.

## 1. Introduction

The increasing occurrence of emerging contaminants in aquatic environments has raised significant concerns regarding their potential impacts on aquatic biota. Coastal and estuarine ecosystems, in particular, act as sinks for a wide range of pollutants due to their proximity to anthropogenic activities, making resident organisms especially vulnerable [1,2,3]. Among these, benthic invertebrates are particularly relevant, as they are continuously exposed to contaminants through both sediment and water [4,5]. Given their ecological importance in nutrient cycling, sediment bioturbation, and trophic interactions, alterations in benthic communities may have consequences for ecosystem functioning [6,7].

Among emerging contaminants, bisphenol A (BPA) and lithium (Li) have received increasing attention due to their widespread industrial use, growing environmental occurrence, and potential effects [8,9]. BPA is widely used in the manufacture of polycarbonate plastics and epoxy resins and is recognized as an endocrine-disrupting compound that can interfere with the endocrine systems of non-target organisms, even at environmentally relevant concentrations [10,11]. BPA and Li have been widely detected in marine and estuarine environments, with BPA concentrations ranging from <0.5 to 670 ng/g dry weight in saline sediments from Europe [12] while Li concentrations have been reported from 21.84 to 146.22 μg/g in sediments from the coastal zone of Malaysia [13]. The environmental occurrence of Li has increased substantially in recent years due to the rapid expansion of lithium-ion battery production, driven by rising demand for electronic devices and electric vehicles. Accordingly, elevated Li concentrations have been reported in wastewater treatment plant effluents and surface waters worldwide [14,15]. Although BPA and Li exhibit distinct mechanisms of toxicity, both have been shown to affect physiological processes in aquatic organisms, including growth, reproduction, metabolism, and oxidative balance [16,17]. In natural environments, organisms are rarely exposed to individual contaminants but instead experience complex chemical mixtures. Interactions among contaminants may result in additive, synergistic, or antagonistic effects, making ecological risk prediction more challenging [18,19,20].

The estuarine polychaete *Hediste diversicolor* (O.F. Müller, 1776) is widely used in ecotoxicological studies due to its broad European distribution, its ecological relevance as a bioturbator and omnivorous deposit feeder, and its robustness under laboratory conditions [21,22,23]. Furthermore, its regenerative capacity has been proposed as a sensitive endpoint for evaluating the sublethal effects of contaminants [24,25]. Following posterior amputation, regeneration involves a coordinated sequence of wound healing, cell dedifferentiation, proliferation, differentiation, and tissue morphogenesis, processes that require substantial energetic investment and are highly dependent on the physiological condition of the organism [26]. Consequently, contaminants capable of disrupting energy metabolism, endocrine regulation, or cellular homeostasis may impair regenerative performance before severe adverse effects become evident.

Previous studies have demonstrated that regeneration in *H. diversicolor* is sensitive to different classes of emerging contaminants, such as graphene oxide nanosheets, polymethylmethacrylate, and polystyrene nanoparticles [24,25,27] (Table 1). Although the effects of BPA and Li have been investigated in some aquatic invertebrates, available information for benthic polychaetes remains limited, particularly regarding regenerative responses. Furthermore, to the best of our knowledge, no studies have evaluated the individual or combined effects of BPA and Li on the regenerative capacity of *H. diversicolor*. This gap is especially relevant given that organisms in natural environments are rarely exposed to single contaminants, but rather to complex mixtures. Therefore, understanding the effects of combined exposures is essential for improving the ecological relevance of risk assessments.

The present study aimed to evaluate the effects of exposure to BPA, Li, and their combination on the regenerative capacity of *H. diversicolor*, providing new insights into the sublethal impacts of these emerging contaminants and their combined toxicity in estuarine environments.

## 2. Materials and Methods

### 2.1. Chemicals

Bisphenol A (BPA, purity > 99%, CAS RN: 80-05-7) was purchased from TCI (Tokyo, Japan). A stock solution of 5 mg/L was prepared to obtain the concentrations of 135, 45, and 15 μg/kg of sediment, and another stock solution of 0.5 mg/L was prepared to obtain the concentrations of 5 and 1.7 μg/kg of sediment. Lithium Chloride (LiCl, CAS 7447-41-8) was purchased from Merck (Darmstadt, Germany). A stock solution of 10 g/L was prepared to obtain concentrations of 2, 5, 12.5, 31.3, and 78.13 mg/kg of sediment.

### 2.2. Test Organisms

Polychaetes were collected from Ria de Aveiro (40.634251, −8.663301) and transported to the laboratory [30]. Upon arrival, organisms were acclimated for two weeks in artificial seawater (ASW) at a salinity of 28 and pH 8.0, using clean sediment in a 3:1 ratio (sediment: water), under continuous aeration at a constant temperature of 16 ± 1 °C. Sediment was obtained from a low-contamination area near the sampling site and sieved in the laboratory through a 1 mm mesh to remove coarse particles and debris [30]. During the acclimation period, polychaetes were fed three times per week with 10 mg of commercial fish food per individual (46.0% protein and 11% lipids), and the water was partially renewed weekly [34,35].

### 2.3. Experimental Setup

Selected polychaetes (90 individuals per experimental condition) were randomly assigned to the following experimental conditions: a) 0, 1.7, 5, 15, 45, and 135 μg of BPA per kg of sediment; b) 0, 2, 5, 12.5, 31.3, and 78.13 mg of Li per kg of sediment. The concentrations selected for the mixture exposure corresponded to the BPA and Li treatments that produced the strongest effects on regeneration in the individual exposure assays; therefore, c) 0, 12.5 mg/kg Li + 5 μg/kg BPA, and 12.5 mg/kg + 45 μg/kg BPA, all with 3 replicates per condition, with 5 organisms each, using sediment spiking [36]. The BPA concentrations were based on literature data reporting BPA concentrations in marine sediments (μg/kg dry weight) [37], whereas Li concentrations (mg/kg dry weight) were selected based on lithium levels previously quantified in sediments from Ria de Aveiro. All exposure concentrations refer to nominal sediment concentrations, as BPA and Li concentrations were not analytically determined in the sediment. The tested concentrations were prepared by adding the appropriate amount of BPA and LiCl stock solution, or only ASW (control), to each beaker containing sediment. Afterward, the sediment was thoroughly homogenized for approximately 10 min. Afterward, ASW was carefully added to avoid disturbing the sediment surface. The experiments were conducted in 1.5 L glass aquaria containing sediment and ASW at a 1:3 (*v*/*v*) ratio, under continuous aeration, with temperature maintained at 16 ± 1 °C, salinity at 28, and pH 8.0, with aeration. Aquaria were allowed to stabilize for one day before introducing organisms. At the start of the exposure period, adult individuals were anesthetized on ice, and the last 15 posterior segments were amputated [27]. Five organisms were kept per glass aquarium under controlled conditions and fed three times per week throughout the experiment, with water renewal once a week. After 21 days, the number of regenerated segments, the width of the regenerated body part, and the width of the last non-regenerating segment were recorded for each individual by visual inspection under a stereomicroscope [38]. Regeneration success was expressed as the percentage of regeneration, calculated as the ratio of the width of newly regenerated segments to that of the original segments before amputation, multiplied by 100 [38].

### 2.4. Statistical Analysis

Regenerating capacity (number of regenerated segments and % of regenerated segments) was examined using permutational multivariate analysis of variance, implemented in PERMANOVA+ within PRIMER v6 [39]. The aquarium was considered the experimental unit (*n* = 3 independent aquaria per treatment), whereas the five organisms maintained within each aquarium were regarded as subsamples. Therefore, individual measurements were averaged within each aquarium before statistical analyses. For each descriptor, differences among treatments were tested and then further explored. The analysis used unrestricted permutations of the raw data, with up to 9999 iterations. Pairwise differences between experimental conditions were evaluated with the Monte Carlo test to obtain specific *p*-values. Statistical significance was set at *p* < 0.05. All descriptors were analyzed under a one-way hierarchical design. The null hypothesis tested was that no significant differences exist among exposure concentrations within each contaminant treatment (BPA, LiCl, and mixture), with the number and percentage of segments analyzed separately.

## 3. Results

### 3.1. BPA Exposure

**Figure 1:**
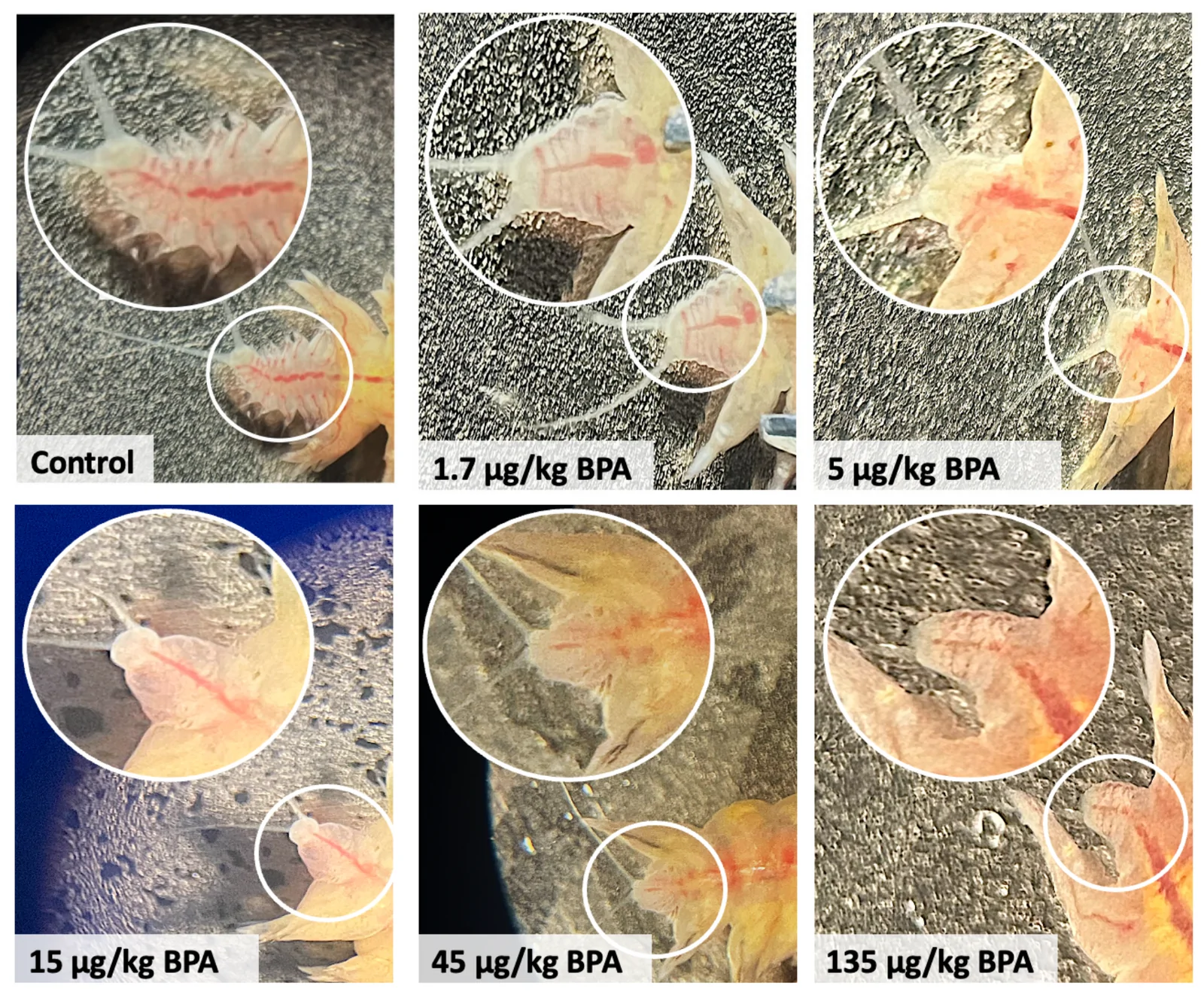
Representative photographs showing the extent of posterior regeneration in *Hediste diversicolor* after 21 days of exposure to BPA (0, 1.7, 5, 15, 45 and 135 µg/kg). Insets show higher-magnification views of the circled regenerated region.

**Figure 2:**
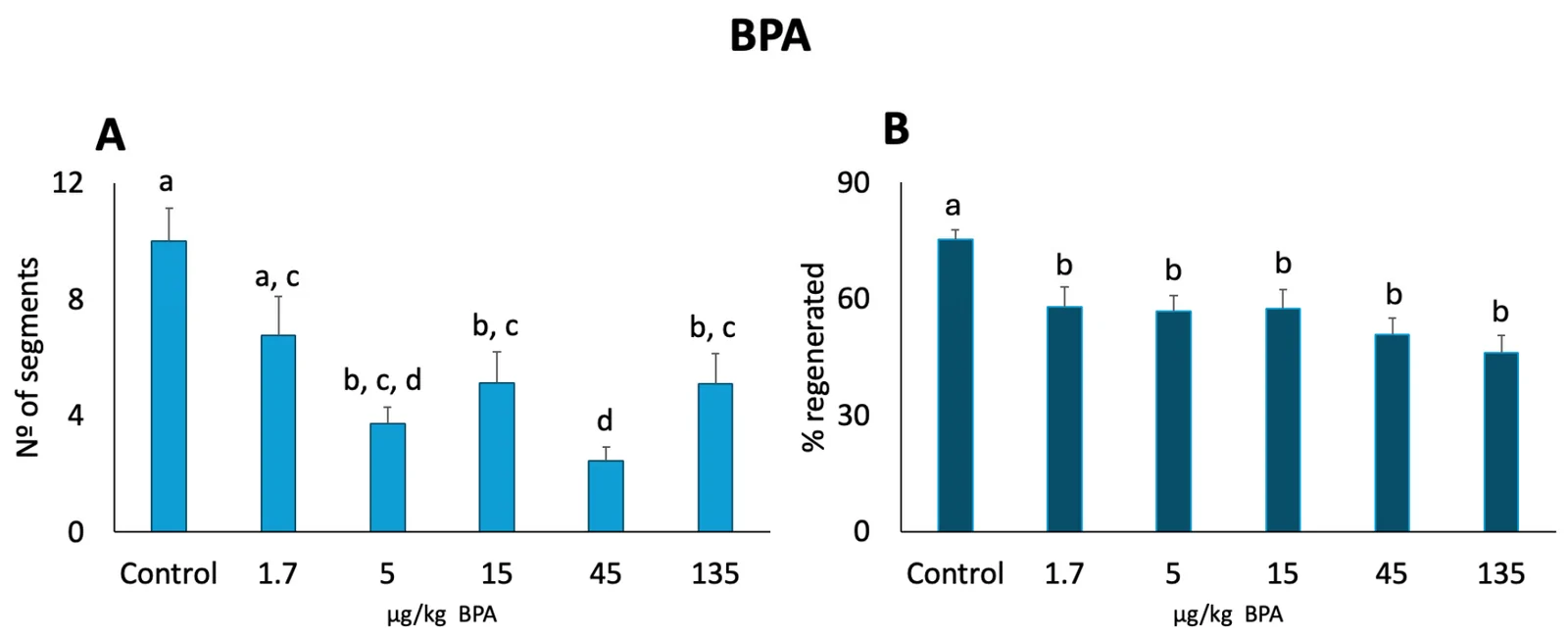
Posterior regenerative capacity of *Hediste diversicolor* after 21 days of BPA (Control-0, 1.7, 5, 15, 45 and 135 µg/kg) exposure. (**A**) Number of regenerated segments (light blue bars) and (**B**) regeneration percentage (dark blue bars), presented as means ± standard deviations. Different lowercase letters indicate significant differences between treatments (*p* < 0.05).

Polychaetes exposed to BPA treatments showed a reduction in the number of regenerated segments in exposed organisms, with more pronounced effects at 5 and 45 μg/kg (Figure 1 and Figure 2A,B). Regarding the percentage of regenerated segments, a significant decrease was observed in all treatments compared with the control, with the lowest values recorded at 135 μg/kg.

### 3.2. Li Exposure

**Figure 3:**
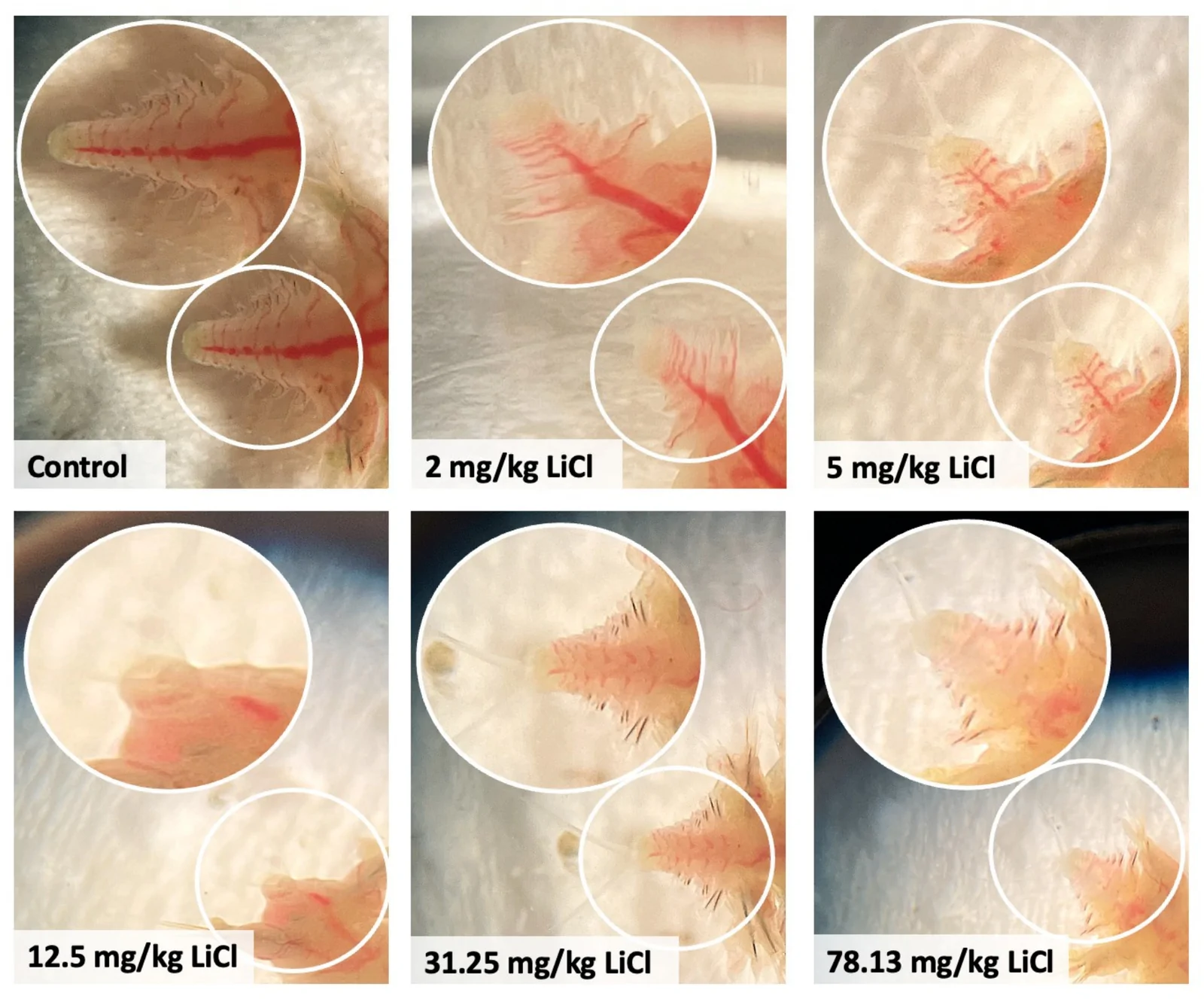
Representative photographs showing the extent of posterior regeneration in *Hediste diversicolor* after 21 days of exposure to Li (0, 2, 5, 12.5, 31.3 and 78.13 mg/kg). Insets show higher-magnification views of the circled regenerated region.

**Figure 4:**
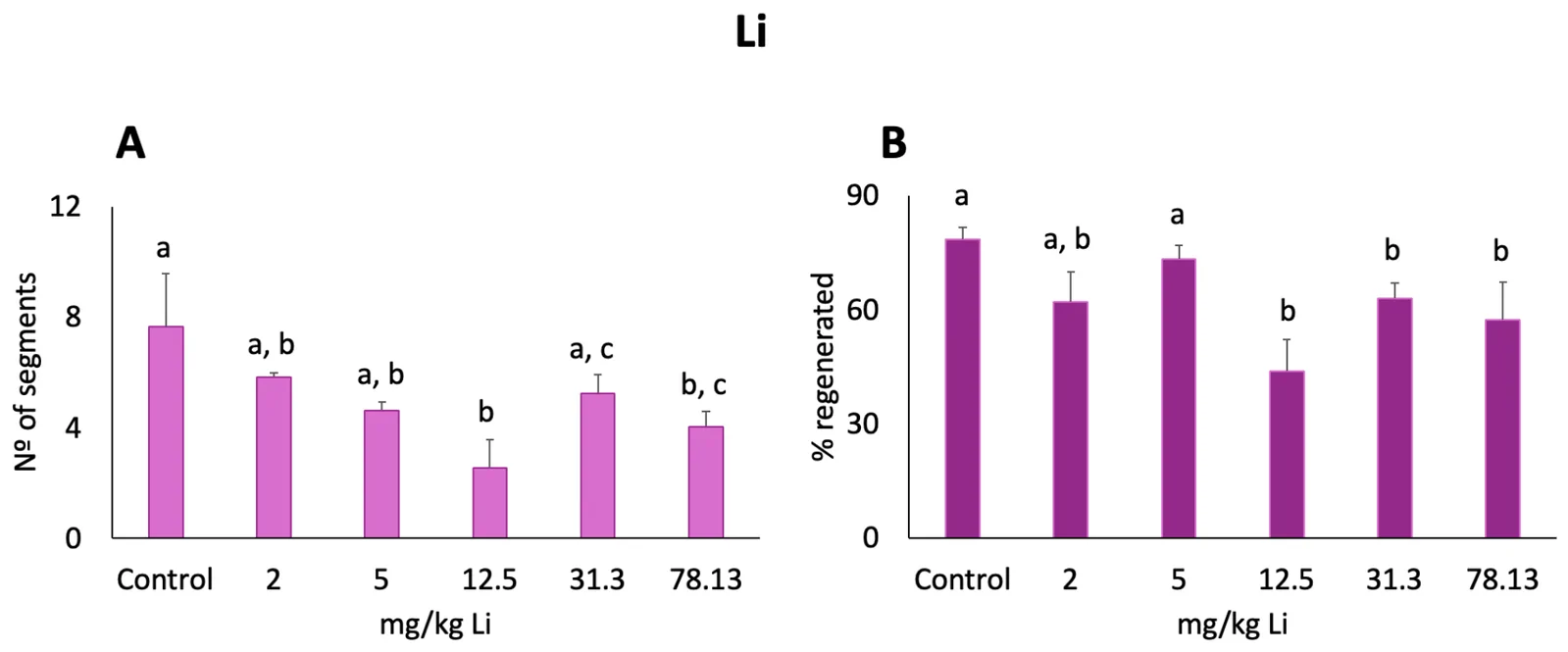
Posterior regenerative capacity of *Hediste diversicolor* after 21 days of Li (Control-0, 2, 5, 12.5, 31.3 and 78.13 mg/kg) exposure. (**A**) Number of regenerated segments (light pink bars) and (**B**) regeneration percentage (dark pink bars), presented as means ± standard deviations. Different lowercase letters indicate significant differences between treatments (*p* < 0.05).

Posterior regenerative capacity of *H. diversicolor* was affected by exposure to Li, both in the number and percentage of regenerated segments, and the response did not follow a clear concentration-dependent pattern (Figure 3 and Figure 4A,B). The number of regenerated segments was lower in organisms exposed to 5 and 12.5 mg/kg, with the lowest values at 12.5 mg/kg. A comparable pattern was observed for the percentage of regenerated segments, with the lowest values recorded at 2 and 12.5 mg/kg, while the remaining concentrations exhibited less pronounced effects.

### 3.3. BPA + Li Exposure

**Figure 5:**
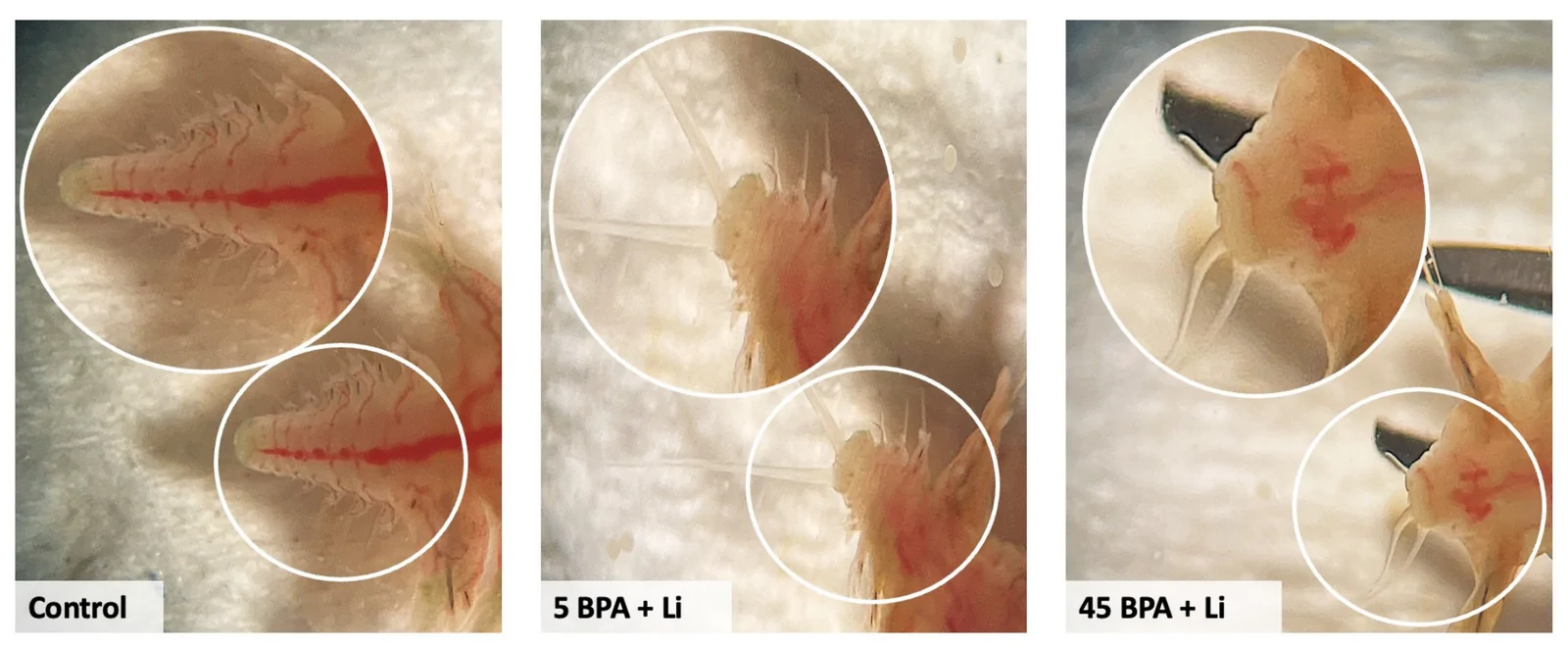
Representative photographs showing the extent of posterior regeneration in *Hediste diversicolor* after 21 days of exposure to BPA + Li (Control-0; 12.5 mg/kg Li + 5 µg/kg BPA; 12.5 mg/kg Li + 45 µg/kg BPA). Insets show higher-magnification views of the circled regenerated region.

**Figure 6:**
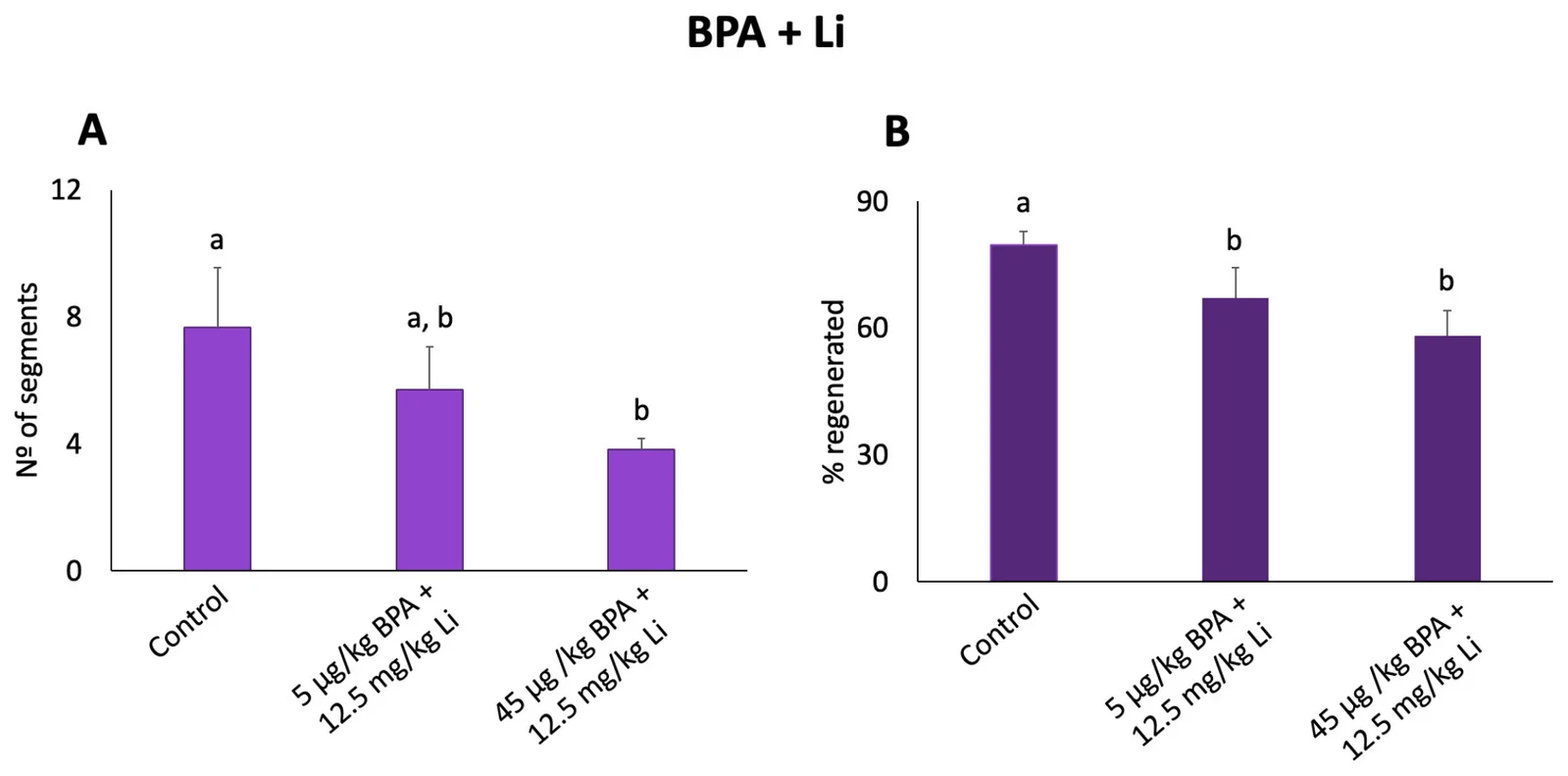
Regenerative capacity of *Hediste diversicolor* after 21 days of combined exposure to BPA and Li (Control-0; 5 µg/kg BPA + 12.5 mg/kg Li; 45 µg/kg BPA + 12.5 mg/kg Li). (**A**) Number of regenerated segments (light purple bars) and (**B**) regeneration percentage (dark purple bars), presented as means ± standard deviations. Different lowercase letters indicate significant differences between treatments (*p* < 0.05).

Organisms exposed to BPA + Li showed lower values of both the number and percentage of regenerated segments compared to the control group (Figure 5 and Figure 6A,B). Among the tested treatments, organisms exposed to 45 µg/kg BPA + 12.5 mg/kg Li exhibited the lowest regenerative ability, showing a significant decrease in the number and percentage of regenerated segments. The mixture containing 5 µg/kg BPA + 12.5 mg/kg Li also had a decrease in the number and percentage of regenerated segments, but it was less pronounced when compared to 45 µg/kg BPA + 12.5 mg/kg Li. The percentage of regenerated segments did not differ significantly between mixture treatments and the respective BPA and Li individual exposures (Table 2).

## 4. Discussion

The present study investigated the effects of BPA, Li, and their combination on the regenerative capacity of *H. diversicolor*, using the number and the percentage of regenerated segments as endpoints. This study showed that exposure to these contaminants negatively affected the regenerative capacity of *H. diversicolor*, as evidenced by the consistent reduction in both the number and percentage of regenerated segments.

For BPA exposure, a clear reduction in regeneration was observed across all treatments, with more pronounced effects at intermediate concentrations (5 and 45 μg/kg). This pattern suggests the absence of a clear concentration-dependent response and may suggest a possible non-monotonic dose–response relationship, a phenomenon commonly reported for endocrine-disrupting compounds such as BPA [40]. The strongest effects were observed at environmentally relevant intermediate concentrations. These findings highlight the ecological relevance of BPA exposure, as adverse biological effects may occur even at concentrations commonly detected in aquatic environments [37]. Moreover, the stronger effects observed at intermediate concentrations suggest that the relationship between exposure concentration and regenerative response may not be strictly dose-dependent. Similar responses have been reported for endocrine-disrupting compounds, in which biological effects do not always increase in proportion to concentration. Previous studies have suggested that non-monotonic responses may be associated with mechanisms such as differential receptor activation, endocrine feedback loops, and compensatory physiological responses [40,41]. The impairment of regeneration following BPA exposure suggests that this contaminant may interfere with fundamental cellular and physiological processes required for tissue repair [42]. BPA is widely recognized as an endocrine-disrupting compound, and previous studies have suggested that interference with hormonal signaling pathways may affect processes such as cell proliferation and differentiation [43], which are important for successful regeneration. Although oxidative stress was not directly measured in the present study, previous studies have consistently shown that BPA induces oxidative damage in marine invertebrates through increased ROS production, lipid peroxidation, and impairment of antioxidant defenses [44,45]. These alterations may potentially compromise the cellular processes required for wound healing and tissue regeneration. In addition to endocrine disruption and oxidative stress, BPA has also been reported to induce neurotoxic effects, including structural alterations in neuronal tissues, characterized by distortion of neuronal cell bodies and varying degrees of pyknosis [45]. Such effects could further compromise wound healing and tissue repair through damage to cellular macromolecules, including proteins, lipids, and DNA, while persistent inflammatory responses may further impair tissue regeneration and recovery [46,47]. In addition, successful regeneration requires coordinated cellular proliferation, differentiation, and tissue remodeling, processes that can be disrupted by neurotoxic effects and alterations in cellular signaling pathways. Previous studies using polychaetes, such as *Diopatra neapolitana* [29,31], have demonstrated that exposure to contaminants like arsenic and graphene oxide nanosheets induces oxidative stress and physiological disruption, resulting in reduced regenerative performance. Overall, the available evidence suggests that BPA-induced impairment of regeneration may be associated with multiple mechanisms, including endocrine disruption, oxidative stress, and neurotoxicity, although these processes were not directly assessed in the present study.

Similarly, exposure to Li decreased both the number and percentage of regenerated segments. The highest reduction in regeneration was observed at an intermediate concentration (12.5 mg/kg), suggesting that regenerative responses may not increase proportionally with Li exposure. Further studies are needed to clarify the mechanisms underlying this pattern. Regeneration is a physiologically demanding process that requires substantial energy investment to support cell proliferation, differentiation, tissue remodeling, and morphogenesis [48]. Under contaminant exposure, organisms may redirect energy reserves from growth and tissue repair towards detoxification processes, antioxidant defenses, and the maintenance of cellular homeostasis [49]. Consequently, reduced regenerative performance could potentially reflect an alteration in energy allocation rather than a direct inhibition of regenerative mechanisms [50]. These findings may indicate that Li exposure could affect physiological processes such as energy metabolism and cellular homeostasis. Supporting this hypothesis, Duan et al. (2025) [51] observed that Li exposure affected gsk-3β expression in *Daphnia magna*, exposed to different Li concentrations (8.34 μg/L, 83.44 μg/L, and 834.41 μg/L), leading to disturbances in mitochondrial dynamics and promoting ROS accumulation and oxidative stress. Similar effects have been reported in other aquatic invertebrates. In *Mytilus galloprovincialis*, Li exposure (0.1, 1, and 10 mg/L of Li for 21 days) induced neurotoxic effects, histopathological alterations, and cellular and tissue damage [52]. Additional studies with the gastropod *Tritia neritea* [53] and the bivalve *M. galloprovincialis* [54] have also reported Li-induced oxidative stress and disrupted cellular homeostasis. Therefore, similar to BPA, the impairment of regeneration observed following Li exposure may suggest an association with oxidative stress and the resulting damage to physiological and cellular processes involved in tissue repair [46,47]. In addition, the energetic demands associated with regeneration may make this process particularly sensitive to physiological disturbances caused by Li exposure. However, these processes were not directly assessed in the present study and therefore remain potential explanations for the observed effects.

When organisms were exposed to the BPA and Li mixture, a reduction in regenerative performance was also observed, with the lowest regeneration observed in organisms exposed to 45 µg/Kg BPA + 12.5 mg/kg Li. However, regeneration in mixture-exposed organisms was generally comparable to, or even higher than, individual exposures, suggesting that combined exposure did not exacerbate the effects of either contaminant (Table 2). These results may suggest that co-exposure did not exacerbate the effects observed under individual exposures. Previous studies have shown that the toxicity of BPA in combination with other contaminants may vary depending on the chemical properties of the mixture and the exposure conditions. For example, Yang et al. [55] reported that the interactions between BPA and heavy metals depended on both the metal involved and the exposure duration. Under acute exposure, BPA (0.62 mg/L) and Cd (13.27 mg/L) exhibited antagonistic interactions, whereas mixtures of BPA with Cr (1.87 mg/L), Pb (7.27 mg/L), Hg (0.94 mg/L), Ni (5.41 mg/L), and As (3.60 mg/L) generally displayed additive effects [55]. In contrast, chronic exposures were predominantly characterized by additive or synergistic interactions [55]. These findings demonstrate that co-exposure does not necessarily result in enhanced toxicity, as contaminant interactions can substantially modify the overall biological response [55]. Antagonistic interactions may arise from several mechanisms, including competition for uptake sites, reduced bioavailability of one contaminant in the presence of another, activation of common detoxification pathways, or compensatory physiological responses that mitigate toxic effects [56,57]. Similar interaction patterns have been described for co-exposure scenarios involving metals and nanoparticles, where changes in contaminant adsorption, accumulation, and bioavailability frequently modify the overall toxic response [56,57]. Such interactions may alter contaminant uptake and toxicity, ultimately reducing their biological impact when compared with single-compound exposure. It should also be noted that only two BPA-Li mixture combinations were tested, selected based on the strongest responses observed during individual exposures. Therefore, although the present results provide evidence regarding the combined effects of BPA and Li, a full factorial design using a wide range of BPA and Li concentrations would allow a more comprehensive evaluation of potential interactions between the two contaminants.

A similar trend to our study was observed in *D. magna* exposed to pyriproxyfen and polystyrene nanoplastics, alone and in combination, for 24 h and 21 days [58]. The adverse effects of pyriproxyfen on the growth and reproduction of *D. magna* were attenuated when organisms were simultaneously exposed to polystyrene nanoplastics, suggesting that co-exposure can alter contaminant uptake and toxicity. Although the mechanisms underlying the interaction between BPA and Li remain poorly explored, the reduced toxicity observed under co-exposure may suggest that interactions occurred between the cellular pathways affected by both contaminants. For example, Li has been reported to affect GSK-3β activity, which may contribute to cellular survival pathways and the maintenance of cellular homeostasis, whereas BPA has been associated with cellular stress and apoptotic responses [44,51]. Furthermore, Li may modulate cellular stress responses, potentially counteracting part of the oxidative damage induced by BPA [51,52,53]. Such interactions may suggest a possible contribution to the overall physiological stress experienced by organisms under combined exposure. However, further studies are required to clarify the processes involved. Taken together, these findings suggest that combined exposures do not necessarily result in greater biological impairment than single-compound exposures, as interactions among contaminants may alter their uptake, bioavailability, or mechanisms of action [59,60]. Nevertheless, as organisms in natural environments are typically exposed to complex contaminant mixtures rather than isolated substances, evaluating mixture effects remains essential for improving the ecological relevance of ecotoxicological risk assessments [61,62].

The observed impairment of regeneration across all treatments further highlights the hypothesis that this endpoint is highly sensitive to contaminant exposure. Similar findings have been reported before in polychaetes, where they were exposed to emerging contaminants (Table 1). In a study where *H. diversicolor* was exposed to 0.01, 0.1, 1 and 10 mg/L of graphene oxide nanosheets for 28 days [24], the regenerative capacity was significantly reduced, and a similar pattern occurred when exposed to polymethylmethacrylate nanoplastics [27]. Also, studies performed with *D. neapolitana* have demonstrated that environmental stressors, including contaminants (e.g., graphene oxide [31], carbamazepine and caffeine [63], carbon nanotubes [28]), can delay regeneration and reduce the number of regenerated segments, further supporting the sensitivity of this endpoint to environmental disturbance. Disruption of these processes may therefore provide an early indication of sublethal toxicity before more evident effects, such as mortality, become apparent, highlighting the value of regeneration as a sensitive ecological and ecotoxicological endpoint [38]. The concentrations tested in the present study were selected based on environmentally reported levels. BPA concentrations fell within the range reported for marine and estuarine sediments [42], while Li concentrations were selected based on concentrations reported for European coastal sediments [64] and observations from estuarine sediments from Ria de Aveiro. Therefore, the nominal concentrations tested encompassed ranges that have been reported in environmental sediments. However, sediment concentrations were not analytically verified, and nominal concentrations may not accurately represent the actual or bioavailable exposure experienced by the organisms. Consequently, the environmental relevance of the observed effects should be interpreted with caution. Nevertheless, the results suggest that BPA and Li can interfere with the regenerative capacity of *H. diversicolor* at nominal concentrations comparable to those reported in environmental sediments. Overall, the results of this study highlight the importance of considering both single and combined contaminant exposures when evaluating ecological risks.

## 5. Conclusions

The present study demonstrated that exposure to BPA, Li, and their mixture affected the regenerative capacity of *Hediste diversicolor*, as evidenced by reductions in both the number and percentage of regenerated segments. These findings highlight regeneration as a sensitive endpoint for detecting the sublethal effects of emerging contaminants. Considering the ecological importance of *H. diversicolor* in estuarine ecosystems, impaired regeneration may affect the ability of organisms to recover from injury, with potential consequences for ecosystem functioning. The effects observed under both single and combined exposures further emphasize the need to consider contaminant mixtures when assessing ecological risks.

Overall, this study provides new evidence of the sublethal impacts of BPA and Li on an ecologically relevant estuarine species and supports the use of regenerative capacity as a sensitive and ecologically relevant endpoint in ecotoxicological assessments of marine benthic organisms. Future studies integrating regenerative responses with biochemical and molecular biomarkers will help clarify the mechanisms underlying the observed effects.

## Figures and Tables

**Table 1:** Overview of published studies evaluating regeneration as a biological endpoint in organisms exposed to environmental stressors. Downward arrows indicate a significant increase or decrease in regenerative capacity (*p* < 0.05) compared to the control.

Species	Environmental Stressor	Concentration in Water (W)/Sediment (S)	Time of Exposure	Impact on Regeneration	Authors
*Hediste diversicolor*	Polystyrene (100 nm)	0.0005, 0.005, 0.05, 0.5, and 5.0 mg/L (W)	28 days	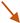 At 0.05, 0.5, and 5.0 mg/L	Silva et al., 2020 [27]
*Hediste diversicolor*	Graphene oxide (GO) nanosheets (~790 nm)	0, 0.01, 0.1, 1 and 10 mg/L (W)	28 days	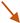 In all treatments	Pires et al., 2022 [24]
*Hediste diversicolor*	Polymethylmethacrylate (~50 nm)	0, 0.5, 5, 50, and 500 μg/L (W)	14 days	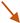 At 5 μg/L	Silva et al., 2023 [25]
*Diopatra neapolitana*	Carbon nanotubes (1680, 2372, 3432 nm)	0.01; 0.10 and 1.00 mg/L (W)	28 days	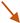 In all treatments	De Marchi et al. 2017 [28]
*Diopatra neapolitana*	Arsenic	0.05, 0.25 and 1.25 mg/L (W)	11, 18 and 28 days	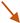 At all treatments	Coppola et al., 2016 [29]
*Diopatra neapolitana*	Sediment contamination-metals	4.31 to 49.73 of total TEs (As, Cd, Cr, Cu, Hg, Ni, Pb) mg/Kg (S)	Until full regeneration	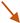 At organisms with higher metal and As bioaccumulation	Pires et al., 2017 [30]
*Diopatra neapolitana*	GO nanomaterial (~200–400 nm)	0.01; 0.10 and 1.00 mg/L (W)	53 days	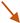 In all treatments	De Marchi et al. 2017 [31]
*Eurythoe complanata*	Crankcase oil	0.3, 1.6, and 3.3% water-soluble fraction (WSF) of used crankcase oil (W)	15 and 21 days	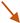 With increasing oil concentration	Nusetti et al. 2005 [32]
*Hermodice carunculate*	Temperature	22 °C and 14 °C (W)	24 weeks	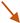 At organisms under 14 °C	Toso et al., 2024 [33]

**Table 2:** Pairwise comparisons between mixture treatments (5 BPA + Li − 5 µg/kg BPA + 12.5 mg/kg Li; 45 BPA + Li − 45 µg/kg BPA + 12.5 mg/kg Li) and individual exposures (Li—12.5 mg/kg Li; 5 BPA—5 µg/kg BPA; 45 BPA—45 µg/kg BPA) based on the percentage of regenerated segments. No significant differences were observed (*p* > 0.05).

Comparison	*p* Value
5 BPA + Li vs. 5 BPA	0.337
5 BPA + Li vs. Li	0.051
45 BPA + Li vs. 45 BPA	0.433
45 BPA + Li vs. Li	0.219

## Data Availability

The raw data supporting the conclusions of this article will be made available by the authors on request.
